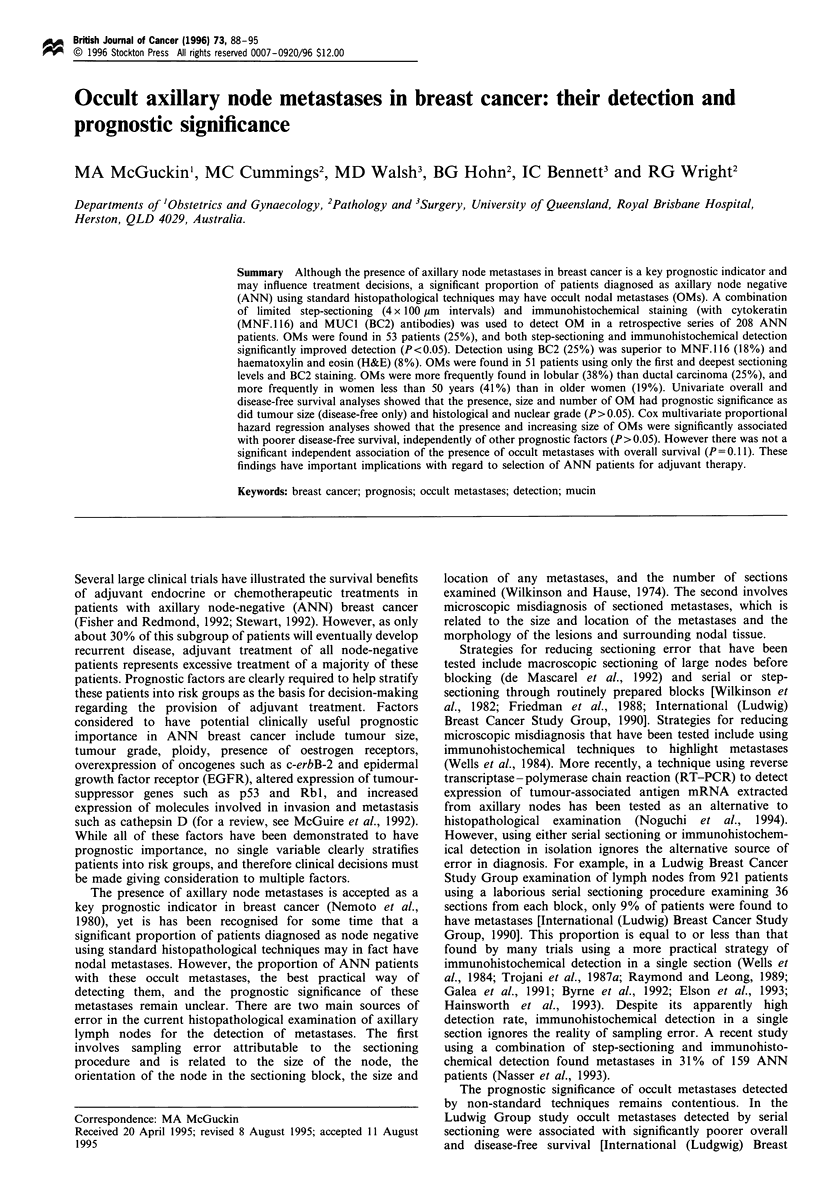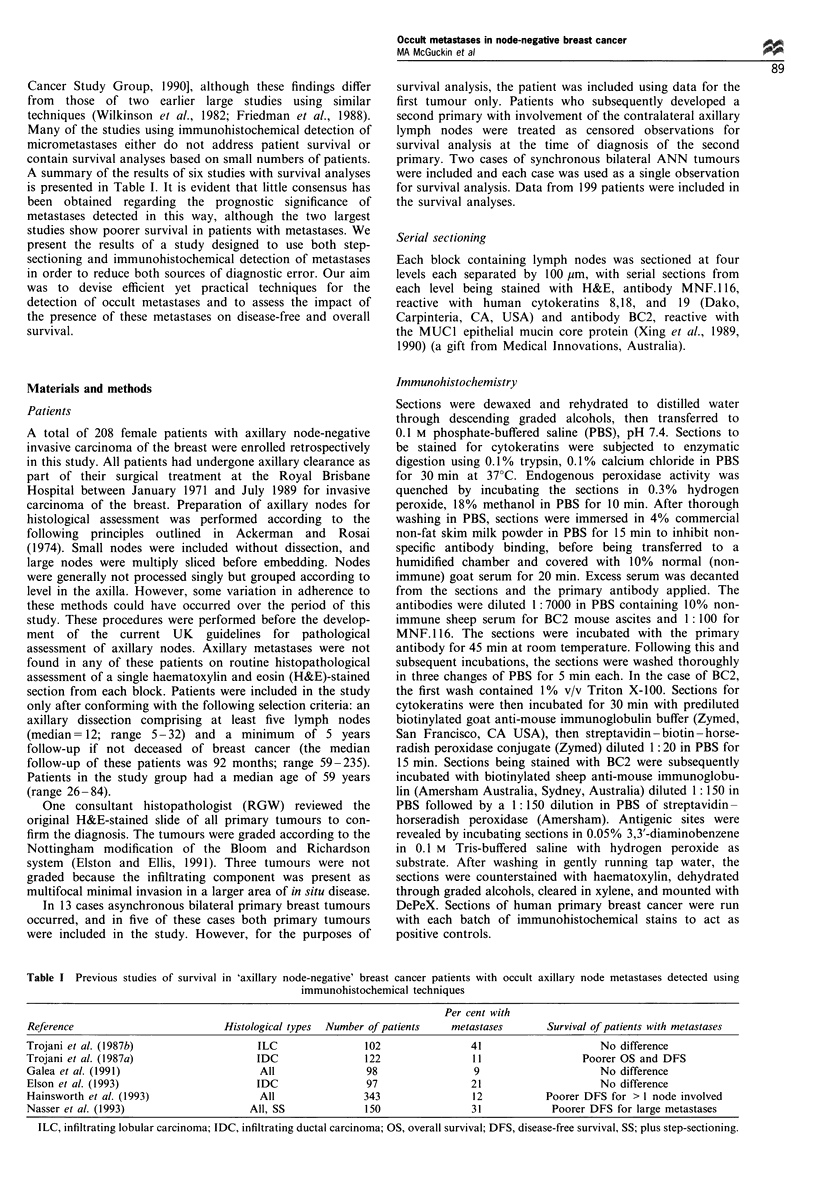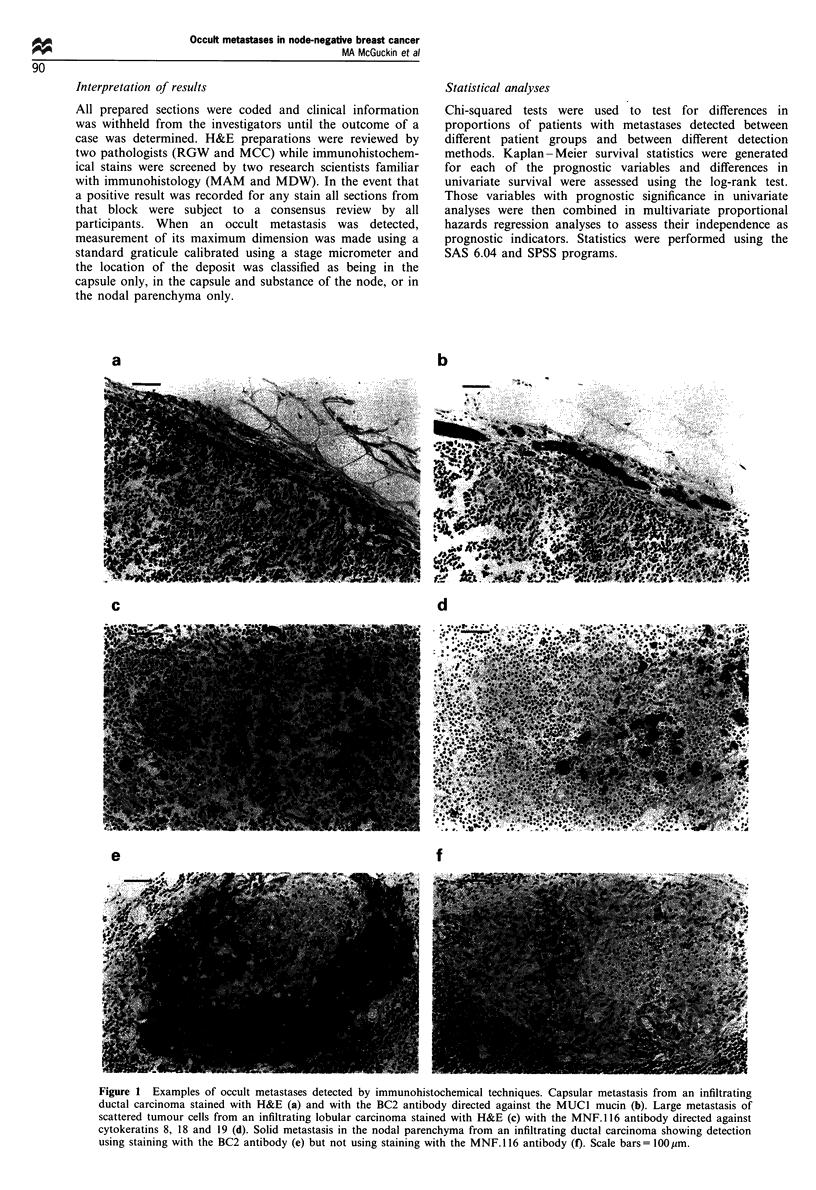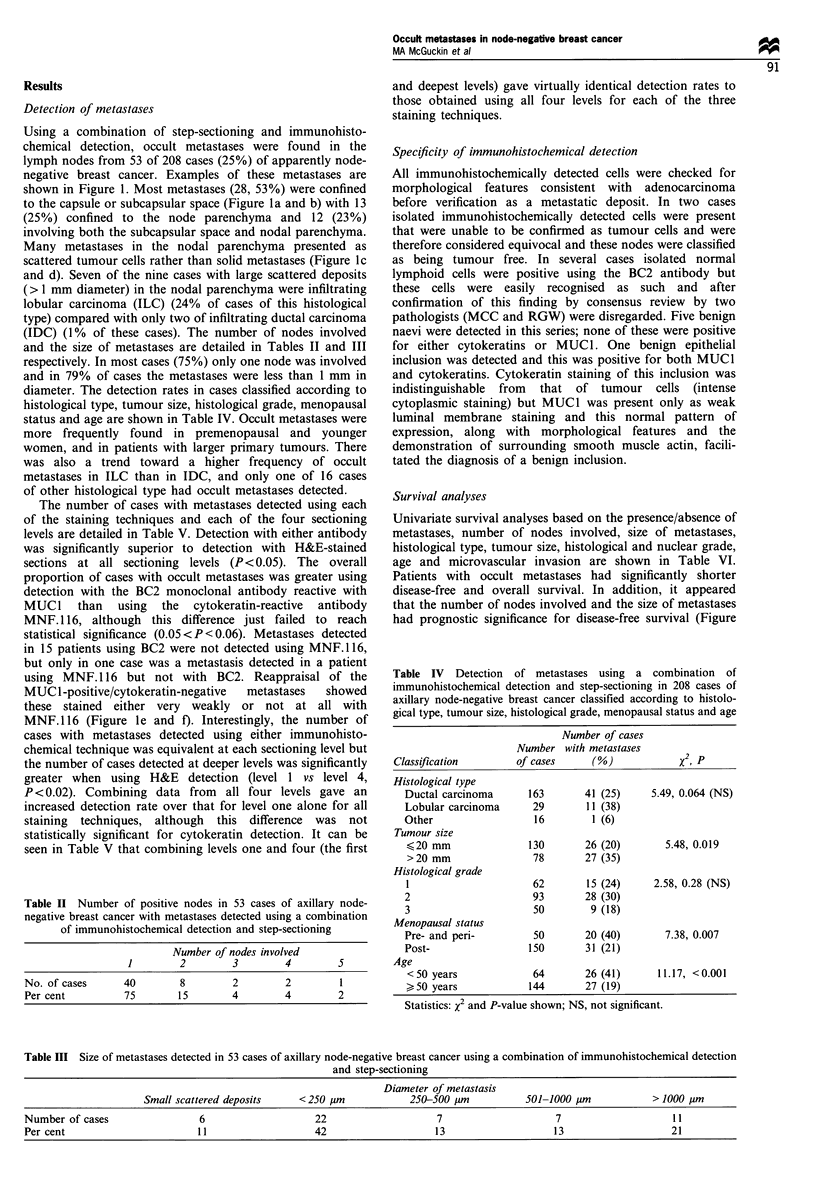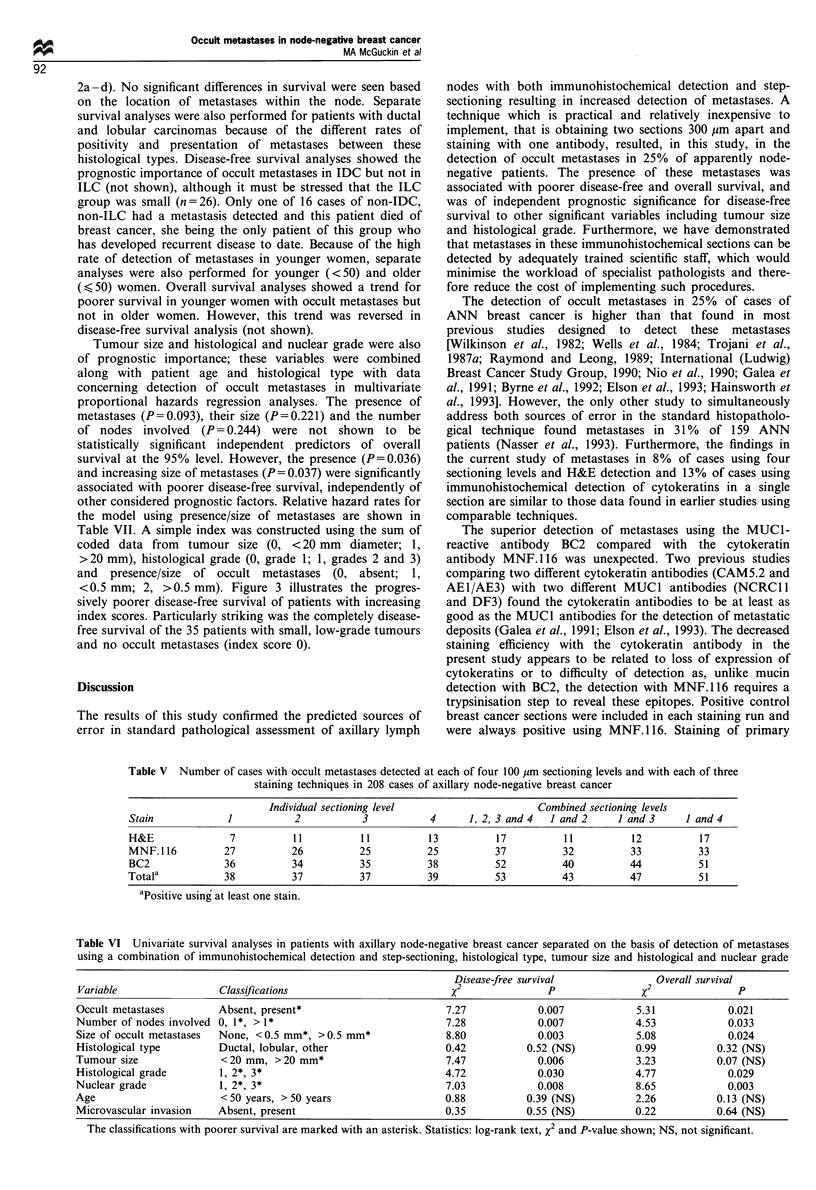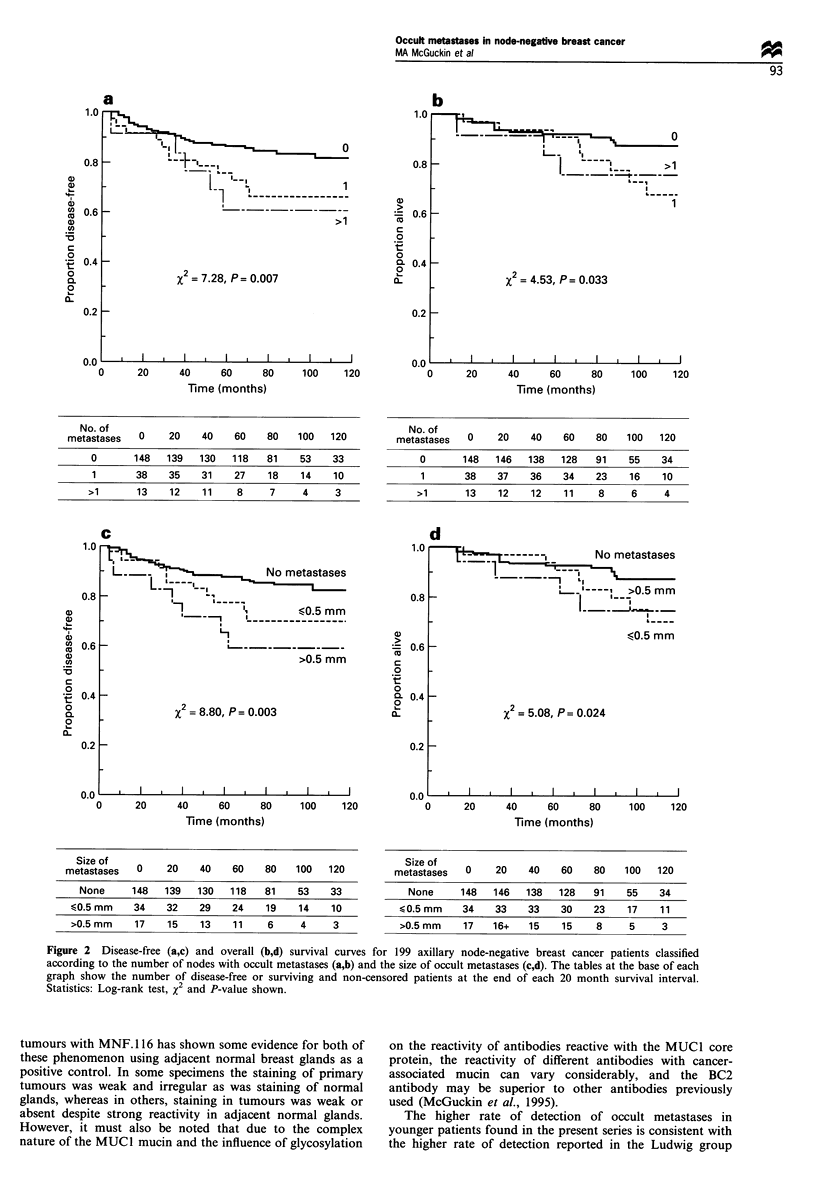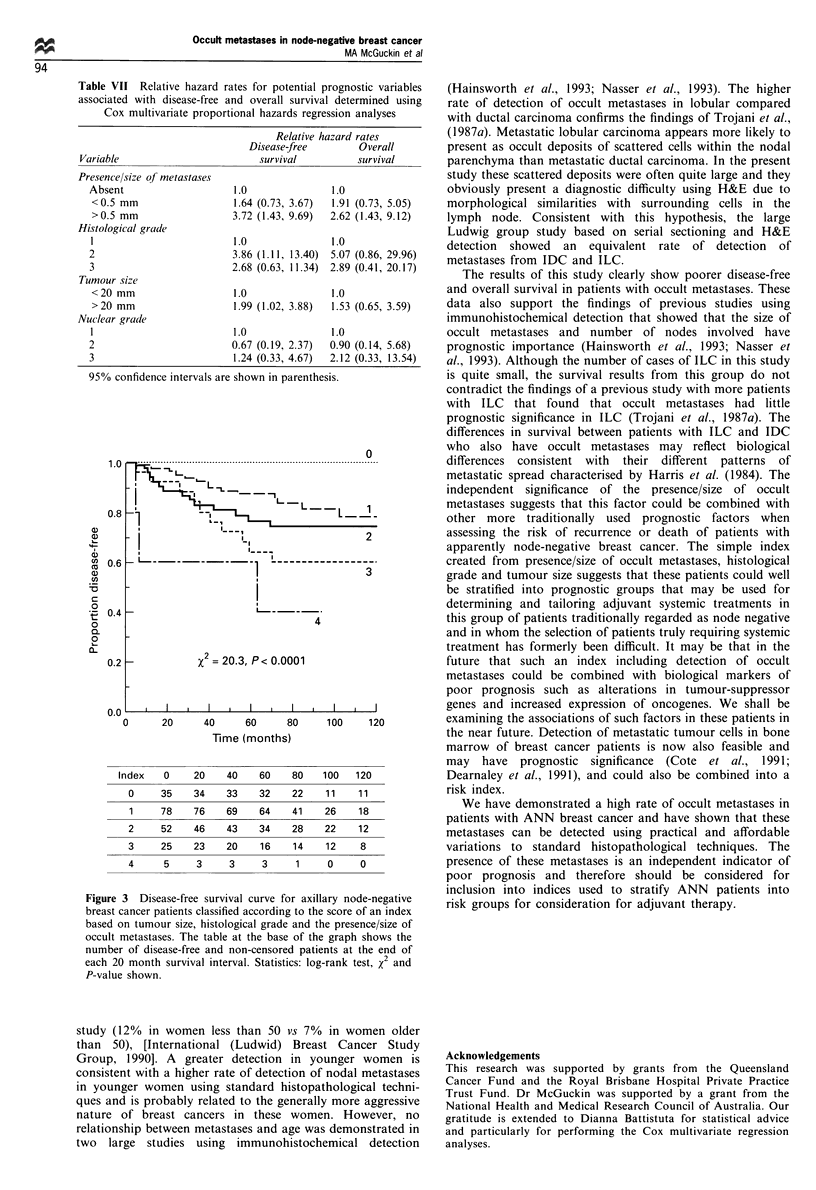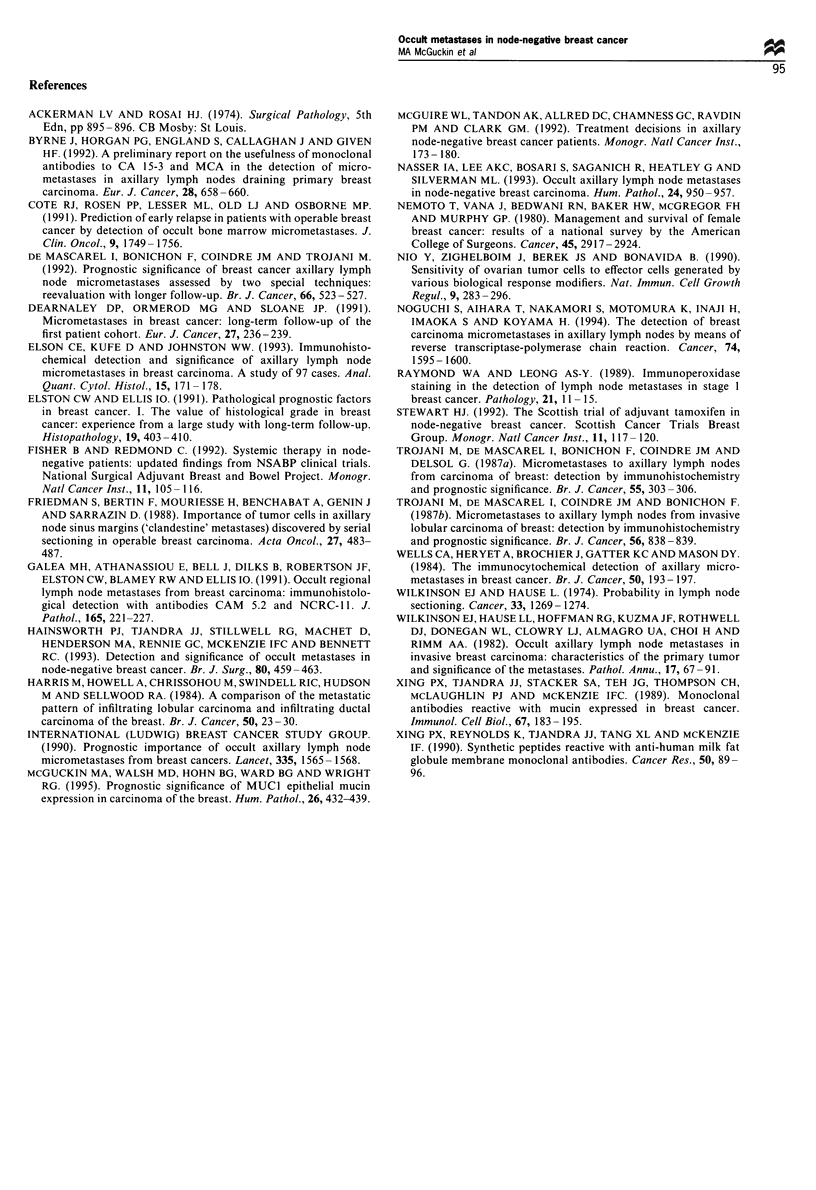# Occult axillary node metastases in breast cancer: their detection and prognostic significance.

**DOI:** 10.1038/bjc.1996.16

**Published:** 1996-01

**Authors:** M. A. McGuckin, M. C. Cummings, M. D. Walsh, B. G. Hohn, I. C. Bennett, R. G. Wright

**Affiliations:** Department of Obstetrics and Gynaecology, University of Queensland, Royal Brisbane Hospital, Herston, Australia.

## Abstract

**Images:**


British Journal of Cancer (1996) 73, 88-95
© 1996 Stockton Press All rights reserved 0007-0920/96 $12.00

### Occult axillary node metastases in breast cancer: their detection and prognostic significance

MA McGuckin[1], MC Cummings[2], MD Walsh[3], BG Hohn[2], IC Bennett[3] and RG Wright[2]

*Departments of [1]Obstetrics and Gynaecology, [2]Pathology and [3]Surgery, University of Queensland, Royal Brisbane Hospital, Herston, QLD 4029, Australia.*

**Summary** Although the presence of axillary node metastases in breast cancer is a key prognostic indicator and may influence treatment decisions, a significant proportion of patients diagnosed as axillary node negative (ANN) using standard histopathological techniques may have occult nodal metastases (OMs). A combination of limited step-sectioning ($4 \times 100\ \mu m$ intervals) and immunohistochemical staining (with cytokeratin (MNF.116) and MUC1 (BC2) antibodies) was used to detect OM in a retrospective series of 208 ANN patients. OMs were found in 53 patients (25%), and both step-sectioning and immunohistochemical detection significantly improved detection ($P<0.05$). Detection using BC2 (25%) was superior to MNF.116 (18%) and haematoxylin and eosin (H&E) (8%). OMs were found in 51 patients using only the first and deepest sectioning levels and BC2 staining. OMs were more frequently found in lobular (38%) than ductal carcinoma (25%), and more frequently in women less than 50 years (41%) than in older women (19%). Univariate overall and disease-free survival analyses showed that the presence, size and number of OM had prognostic significance as did tumour size (disease-free only) and histological and nuclear grade ($P>0.05$). Cox multivariate proportional hazard regression analyses showed that the presence and increasing size of OMs were significantly associated with poorer disease-free survival, independently of other prognostic factors ($P>0.05$). However there was not a significant independent association of the presence of occult metastases with overall survival ($P=0.11$). These findings have important implications with regard to selection of ANN patients for adjuvant therapy.

**Keywords:** breast cancer; prognosis; occult metastases; detection; mucin

Several large clinical trials have illustrated the survival benefits of adjuvant endocrine or chemotherapeutic treatments in patients with axillary node-negative (ANN) breast cancer (Fisher and Redmond, 1992; Stewart, 1992). However, as only about 30% of this subgroup of patients will eventually develop recurrent disease, adjuvant treatment of all node-negative patients represents excessive treatment of a majority of these patients. Prognostic factors are clearly required to help stratify these patients into risk groups as the basis for decision-making regarding the provision of adjuvant treatment. Factors considered to have potential clinically useful prognostic importance in ANN breast cancer include tumour size, tumour grade, ploidy, presence of oestrogen receptors, overexpression of oncogenes such as c-*erb*B-2 and epidermal growth factor receptor (EGFR), altered expression of tumour-suppressor genes such as p53 and Rb1, and increased expression of molecules involved in invasion and metastasis such as cathepsin D (for a review, see McGuire *et al.*, 1992). While all of these factors have been demonstrated to have prognostic importance, no single variable clearly stratifies patients into risk groups, and therefore clinical decisions must be made giving consideration to multiple factors.

The presence of axillary node metastases is accepted as a key prognostic indicator in breast cancer (Nemoto *et al.*, 1980), yet is has been recognised for some time that a significant proportion of patients diagnosed as node negative using standard histopathological techniques may in fact have nodal metastases. However, the proportion of ANN patients with these occult metastases, the best practical way of detecting them, and the prognostic significance of these metastases remain unclear. There are two main sources of error in the current histopathological examination of axillary lymph nodes for the detection of metastases. The first involves sampling error attributable to the sectioning procedure and is related to the size of the node, the orientation of the node in the sectioning block, the size and location of any metastases, and the number of sections examined (Wilkinson and Hause, 1974). The second involves microscopic misdiagnosis of sectioned metastases, which is related to the size and location of the metastases and the morphology of the lesions and surrounding nodal tissue.

Strategies for reducing sectioning error that have been tested include macroscopic sectioning of large nodes before blocking (de Mascarel *et al.*, 1992) and serial or step-sectioning through routinely prepared blocks [Wilkinson *et al.*, 1982; Friedman *et al.*, 1988; International (Ludwig) Breast Cancer Study Group, 1990]. Strategies for reducing microscopic misdiagnosis that have been tested include using immunohistochemical techniques to highlight metastases (Wells *et al.*, 1984). More recently, a technique using reverse transcriptase-polymerase chain reaction (RT-PCR) to detect expression of tumour-associated antigen mRNA extracted from axillary nodes has been tested as an alternative to histopathological examination (Noguchi *et al.*, 1994). However, using either serial sectioning or immunohistochemical detection in isolation ignores the alternative source of error in diagnosis. For example, in a Ludwig Breast Cancer Study Group examination of lymph nodes from 921 patients using a laborious serial sectioning procedure examining 36 sections from each block, only 9% of patients were found to have metastases [International (Ludwig) Breast Cancer Study Group, 1990]. This proportion is equal to or less than that found by many trials using a more practical strategy of immunohistochemical detection in a single section (Wells *et al.*, 1984; Trojani *et al.*, 1987*a*; Raymond and Leong, 1989; Galea *et al.*, 1991; Byrne *et al.*, 1992; Elson *et al.*, 1993; Hainsworth *et al.*, 1993). Despite its apparently high detection rate, immunohistochemical detection in a single section ignores the reality of sampling error. A recent study using a combination of step-sectioning and immunohistochemical detection found metastases in 31% of 159 ANN patients (Nasser *et al.*, 1993).

The prognostic significance of occult metastases detected by non-standard techniques remains contentious. In the Ludwig Group study occult metastases detected by serial sectioning were associated with significantly poorer overall and disease-free survival [International (Ludgwig) Breast

Correspondence: MA McGuckin
Received 20 April 1995; revised 8 August 1995; accepted 11 August 1995

Cancer Study Group, 1990], although these findings differ from those of two earlier large studies using similar techniques (Wilkinson *et al.*, 1982; Friedman *et al.*, 1988). Many of the studies using immunohistochemical detection of micrometastases either do not address patient survival or contain survival analyses based on small numbers of patients. A summary of the results of six studies with survival analyses is presented in Table I. It is evident that little consensus has been obtained regarding the prognostic significance of metastases detected in this way, although the two largest studies show poorer survival in patients with metastases. We present the results of a study designed to use both step-sectioning and immunohistochemical detection of metastases in order to reduce both sources of diagnostic error. Our aim was to devise efficient yet practical techniques for the detection of occult metastases and to assess the impact of the presence of these metastases on disease-free and overall survival.

### Materials and methods

### *Patients*

A total of 208 female patients with axillary node-negative invasive carcinoma of the breast were enrolled retrospectively in this study. All patients had undergone axillary clearance as part of their surgical treatment at the Royal Brisbane Hospital between January 1971 and July 1989 for invasive carcinoma of the breast. Preparation of axillary nodes for histological assessment was performed according to the following principles outlined in Ackerman and Rosai (1974). Small nodes were included without dissection, and large nodes were multiply sliced before embedding. Nodes were generally not processed singly but grouped according to level in the axilla. However, some variation in adherence to these methods could have occurred over the period of this study. These procedures were performed before the development of the current UK guidelines for pathological assessment of axillary nodes. Axillary metastases were not found in any of these patients on routine histopathological assessment of a single haematoxylin and eosin (H&E)-stained section from each block. Patients were included in the study only after conforming with the following selection criteria: an axillary dissection comprising at least five lymph nodes (median = 12; range 5–32) and a minimum of 5 years follow-up if not deceased of breast cancer (the median follow-up of these patients was 92 months; range 59–235). Patients in the study group had a median age of 59 years (range 26–84).

One consultant histopathologist (RGW) reviewed the original H&E-stained slide of all primary tumours to confirm the diagnosis. The tumours were graded according to the Nottingham modification of the Bloom and Richardson system (Elston and Ellis, 1991). Three tumours were not graded because the infiltrating component was present as multifocal minimal invasion in a larger area of *in situ* disease.

In 13 cases asynchronous bilateral primary breast tumours occurred, and in five of these cases both primary tumours were included in the study. However, for the purposes of survival analysis, the patient was included using data for the first tumour only. Patients who subsequently developed a second primary with involvement of the contralateral axillary lymph nodes were treated as censored observations for survival analysis at the time of diagnosis of the second primary. Two cases of synchronous bilateral ANN tumours were included and each case was used as a single observation for survival analysis. Data from 199 patients were included in the survival analyses.

### *Serial sectioning*

Each block containing lymph nodes was sectioned at four levels each separated by 100 μm, with serial sections from each level being stained with H&E, antibody MNF.116, reactive with human cytokeratins 8,18, and 19 (Dako, Carpinteria, CA, USA) and antibody BC2, reactive with the MUC1 epithelial mucin core protein (Xing *et al.*, 1989, 1990) (a gift from Medical Innovations, Australia).

### *Immunohistochemistry*

Sections were dewaxed and rehydrated to distilled water through descending graded alcohols, then transferred to 0.1 M phosphate-buffered saline (PBS), pH 7.4. Sections to be stained for cytokeratins were subjected to enzymatic digestion using 0.1% trypsin, 0.1% calcium chloride in PBS for 30 min at 37°C. Endogenous peroxidase activity was quenched by incubating the sections in 0.3% hydrogen peroxide, 18% methanol in PBS for 10 min. After thorough washing in PBS, sections were immersed in 4% commercial non-fat skim milk powder in PBS for 15 min to inhibit non-specific antibody binding, before being transferred to a humidified chamber and covered with 10% normal (non-immune) goat serum for 20 min. Excess serum was decanted from the sections and the primary antibody applied. The antibodies were diluted 1 : 7000 in PBS containing 10% non-immune sheep serum for BC2 mouse ascites and 1 : 100 for MNF.116. The sections were incubated with the primary antibody for 45 min at room temperature. Following this and subsequent incubations, the sections were washed thoroughly in three changes of PBS for 5 min each. In the case of BC2, the first wash contained 1% v/v Triton X-100. Sections for cytokeratins were then incubated for 30 min with prediluted biotinylated goat anti-mouse immunoglobulin buffer (Zymed, San Francisco, CA USA), then streptavidin–biotin–horseradish peroxidase conjugate (Zymed) diluted 1 : 20 in PBS for 15 min. Sections being stained with BC2 were subsequently incubated with biotinylated sheep anti-mouse immunoglobulin (Amersham Australia, Sydney, Australia) diluted 1 : 150 in PBS followed by a 1 : 150 dilution in PBS of streptavidin–horseradish peroxidase (Amersham). Antigenic sites were revealed by incubating sections in 0.05% 3,3′-diaminobenzene in 0.1 M Tris-buffered saline with hydrogen peroxide as substrate. After washing in gently running tap water, the sections were counterstained with haematoxylin, dehydrated through graded alcohols, cleared in xylene, and mounted with DePeX. Sections of human primary breast cancer were run with each batch of immunohistochemical stains to act as positive controls.

**Table I** Previous studies of survival in 'axillary node-negative' breast cancer patients with occult axillary node metastases detected using immunohistochemical techniques

| *Reference* | *Histological types* | *Number of patients* | *Per cent with metastases* | *Survival of patients with metastases* |
|---|---|---|---|---|
| Trojani *et al.* (1987*b*) | ILC | 102 | 41 | No difference |
| Trojani *et al.* (1987*a*) | IDC | 122 | 11 | Poorer OS and DFS |
| Galea *et al.* (1991) | All | 98 | 9 | No difference |
| Elson *et al.* (1993) | IDC | 97 | 21 | No difference |
| Hainsworth *et al.* (1993) | All | 343 | 12 | Poorer DFS for > 1 node involved |
| Nasser *et al.* (1993) | All, SS | 150 | 31 | Poorer DFS for large metastases |

ILC, infiltrating lobular carcinoma; IDC, infiltrating ductal carcinoma; OS, overall survival; DFS, disease-free survival, SS; plus step-sectioning.

*Interpretation of results*

All prepared sections were coded and clinical information was withheld from the investigators until the outcome of a case was determined. H&E preparations were reviewed by two pathologists (RGW and MCC) while immunohistochemical stains were screened by two research scientists familiar with immunohistology (MAM and MDW). In the event that a positive result was recorded for any stain all sections from that block were subject to a consensus review by all participants. When an occult metastasis was detected, measurement of its maximum dimension was made using a standard graticule calibrated using a stage micrometer and the location of the deposit was classified as being in the capsule only, in the capsule and substance of the node, or in the nodal parenchyma only.

*Statistical analyses*

Chi-squared tests were used to test for differences in proportions of patients with metastases detected between different patient groups and between different detection methods. Kaplan–Meier survival statistics were generated for each of the prognostic variables and differences in univariate survival were assessed using the log-rank test. Those variables with prognostic significance in univariate analyses were then combined in multivariate proportional hazards regression analyses to assess their independence as prognostic indicators. Statistics were performed using the SAS 6.04 and SPSS programs.

**Figure 1** Examples of occult metastases detected by immunohistochemical techniques. Capsular metastasis from an infiltrating ductal carcinoma stained with H&E (**a**) and with the BC2 antibody directed against the MUC1 mucin (**b**). Large metastasis of scattered tumour cells from an infiltrating lobular carcinoma stained with H&E (**c**) with the MNF.116 antibody directed against cytokeratins 8, 18 and 19 (**d**). Solid metastasis in the nodal parenchyma from an infiltrating ductal carcinoma showing detection using staining with the BC2 antibody (**e**) but not using staining with the MNF.116 antibody (**f**). Scale bars = 100 $\mu$m.

### Results

*Detection of metastases*

Using a combination of step-sectioning and immunohistochemical detection, occult metastases were found in the lymph nodes from 53 of 208 cases (25%) of apparently node-negative breast cancer. Examples of these metastases are shown in Figure 1. Most metastases (28, 53%) were confined to the capsule or subcapsular space (Figure 1a and b) with 13 (25%) confined to the node parenchyma and 12 (23%) involving both the subcapsular space and nodal parenchyma. Many metastases in the nodal parenchyma presented as scattered tumour cells rather than solid metastases (Figure 1c and d). Seven of the nine cases with large scattered deposits (> 1 mm diameter) in the nodal parenchyma were infiltrating lobular carcinoma (ILC) (24% of cases of this histological type) compared with only two of infiltrating ductal carcinoma (IDC) (1% of these cases). The number of nodes involved and the size of metastases are detailed in Tables II and III respectively. In most cases (75%) only one node was involved and in 79% of cases the metastases were less than 1 mm in diameter. The detection rates in cases classified according to histological type, tumour size, histological grade, menopausal status and age are shown in Table IV. Occult metastases were more frequently found in premenopausal and younger women, and in patients with larger primary tumours. There was also a trend toward a higher frequency of occult metastases in ILC than in IDC, and only one of 16 cases of other histological type had occult metastases detected.

The number of cases with metastases detected using each of the staining techniques and each of the four sectioning levels are detailed in Table V. Detection with either antibody was significantly superior to detection with H&E-stained sections at all sectioning levels ($P<0.05$). The overall proportion of cases with occult metastases was greater using detection with the BC2 monoclonal antibody reactive with MUC1 than using the cytokeratin-reactive antibody MNF.116, although this difference just failed to reach statistical significance ($0.05<P<0.06$). Metastases detected in 15 patients using BC2 were not detected using MNF.116, but only in one case was a metastasis detected in a patient using MNF.116 but not with BC2. Reappraisal of the MUC1-positive/cytokeratin-negative metastases showed these stained either very weakly or not at all with MNF.116 (Figure 1e and f). Interestingly, the number of cases with metastases detected using either immunohistochemical technique was equivalent at each sectioning level but the number of cases detected at deeper levels was significantly greater when using H&E detection (level 1 *vs* level 4, $P<0.02$). Combining data from all four levels gave an increased detection rate over that for level one alone for all staining techniques, although this difference was not statistically significant for cytokeratin detection. It can be seen in Table V that combining levels one and four (the first and deepest levels) gave virtually identical detection rates to those obtained using all four levels for each of the three staining techniques.

*Specificity of immunohistochemical detection*

All immunohistochemically detected cells were checked for morphological features consistent with adenocarcinoma before verification as a metastatic deposit. In two cases isolated immunohistochemically detected cells were present that were unable to be confirmed as tumour cells and were therefore considered equivocal and these nodes were classified as being tumour free. In several cases isolated normal lymphoid cells were positive using the BC2 antibody but these cells were easily recognised as such and after confirmation of this finding by consensus review by two pathologists (MCC and RGW) were disregarded. Five benign naevi were detected in this series; none of these were positive for either cytokeratins or MUC1. One benign epithelial inclusion was detected and this was positive for both MUC1 and cytokeratins. Cytokeratin staining of this inclusion was indistinguishable from that of tumour cells (intense cytoplasmic staining) but MUC1 was present only as weak luminal membrane staining and this normal pattern of expression, along with morphological features and the demonstration of surrounding smooth muscle actin, facilitated the diagnosis of a benign inclusion.

*Survival analyses*

Univariate survival analyses based on the presence/absence of metastases, number of nodes involved, size of metastases, histological type, tumour size, histological and nuclear grade, age and microvascular invasion are shown in Table VI. Patients with occult metastases had significantly shorter disease-free and overall survival. In addition, it appeared that the number of nodes involved and the size of metastases had prognostic significance for disease-free survival (Figure

**Table II** Number of positive nodes in 53 cases of axillary node-negative breast cancer with metastases detected using a combination of immunohistochemical detection and step-sectioning

| | *Number of nodes involved* | | | | |
|---|---|---|---|---|---|
| | *1* | *2* | *3* | *4* | *5* |
| No. of cases | 40 | 8 | 2 | 2 | 1 |
| Per cent | 75 | 15 | 4 | 4 | 2 |

**Table IV** Detection of metastases using a combination of immunohistochemical detection and step-sectioning in 208 cases of axillary node-negative breast cancer classified according to histological type, tumour size, histological grade, menopausal status and age

| *Classification* | *Number of cases* | *Number of cases with metastases (%)* | $\chi^2$, *P* |
|---|---|---|---|
| *Histological type* | | | |
| Ductal carcinoma | 163 | 41 (25) | 5.49, 0.064 (NS) |
| Lobular carcinoma | 29 | 11 (38) | |
| Other | 16 | 1 (6) | |
| *Tumour size* | | | |
| $\leqslant$20 mm | 130 | 26 (20) | 5.48, 0.019 |
| >20 mm | 78 | 27 (35) | |
| *Histological grade* | | | |
| 1 | 62 | 15 (24) | 2.58, 0.28 (NS) |
| 2 | 93 | 28 (30) | |
| 3 | 50 | 9 (18) | |
| *Menopausal status* | | | |
| Pre- and peri- | 50 | 20 (40) | 7.38, 0.007 |
| Post- | 150 | 31 (21) | |
| *Age* | | | |
| <50 years | 64 | 26 (41) | 11.17, <0.001 |
| $\geqslant$50 years | 144 | 27 (19) | |

Statistics: $\chi^2$ and *P*-value shown; NS, not significant.

**Table III** Size of metastases detected in 53 cases of axillary node-negative breast cancer using a combination of immunohistochemical detection and step-sectioning

| | *Diameter of metastasis* | | | | |
|---|---|---|---|---|---|
| | *Small scattered deposits* | *<250 μm* | *250–500 μm* | *501–1000 μm* | *>1000 μm* |
| Number of cases | 6 | 22 | 7 | 7 | 11 |
| Per cent | 11 | 42 | 13 | 13 | 21 |

2a–d). No significant differences in survival were seen based on the location of metastases within the node. Separate survival analyses were also performed for patients with ductal and lobular carcinomas because of the different rates of positivity and presentation of metastases between these histological types. Disease-free survival analyses showed the prognostic importance of occult metastases in IDC but not in ILC (not shown), although it must be stressed that the ILC group was small ($n = 26$). Only one of 16 cases of non-IDC, non-ILC had a metastasis detected and this patient died of breast cancer, she being the only patient of this group who has developed recurrent disease to date. Because of the high rate of detection of metastases in younger women, separate analyses were also performed for younger (<50) and older (⩽50) women. Overall survival analyses showed a trend for poorer survival in younger women with occult metastases but not in older women. However, this trend was reversed in disease-free survival analysis (not shown).

Tumour size and histological and nuclear grade were also of prognostic importance; these variables were combined along with patient age and histological type with data concerning detection of occult metastases in multivariate proportional hazards regression analyses. The presence of metastases ($P = 0.093$), their size ($P = 0.221$) and the number of nodes involved ($P = 0.244$) were not shown to be statistically significant independent predictors of overall survival at the 95% level. However, the presence ($P = 0.036$) and increasing size of metastases ($P = 0.037$) were significantly associated with poorer disease-free survival, independently of other considered prognostic factors. Relative hazard rates for the model using presence/size of metastases are shown in Table VII. A simple index was constructed using the sum of coded data from tumour size (0, <20 mm diameter; 1, >20 mm), histological grade (0, grade 1; 1, grades 2 and 3) and presence/size of occult metastases (0, absent; 1, <0.5 mm; 2, >0.5 mm). Figure 3 illustrates the progressively poorer disease-free survival of patients with increasing index scores. Particularly striking was the completely disease-free survival of the 35 patients with small, low-grade tumours and no occult metastases (index score 0).

### Discussion

The results of this study confirmed the predicted sources of error in standard pathological assessment of axillary lymph nodes with both immunohistochemical detection and step-sectioning resulting in increased detection of metastases. A technique which is practical and relatively inexpensive to implement, that is obtaining two sections 300 μm apart and staining with one antibody, resulted, in this study, in the detection of occult metastases in 25% of apparently node-negative patients. The presence of these metastases was associated with poorer disease-free and overall survival, and was of independent prognostic significance for disease-free survival to other significant variables including tumour size and histological grade. Furthermore, we have demonstrated that metastases in these immunohistochemical sections can be detected by adequately trained scientific staff, which would minimise the workload of specialist pathologists and therefore reduce the cost of implementing such procedures.

The detection of occult metastases in 25% of cases of ANN breast cancer is higher than that found in most previous studies designed to detect these metastases [Wilkinson *et al.*, 1982; Wells *et al.*, 1984; Trojani *et al.*, 1987*a*; Raymond and Leong, 1989; International (Ludwig) Breast Cancer Study Group, 1990; Nio *et al.*, 1990; Galea *et al.*, 1991; Byrne *et al.*, 1992; Elson *et al.*, 1993; Hainsworth *et al.*, 1993]. However, the only other study to simultaneously address both sources of error in the standard histopathological technique found metastases in 31% of 159 ANN patients (Nasser *et al.*, 1993). Furthermore, the findings in the current study of metastases in 8% of cases using four sectioning levels and H&E detection and 13% of cases using immunohistochemical detection of cytokeratins in a single section are similar to those data found in earlier studies using comparable techniques.

The superior detection of metastases using the MUC1-reactive antibody BC2 compared with the cytokeratin antibody MNF.116 was unexpected. Two previous studies comparing two different cytokeratin antibodies (CAM5.2 and AE1/AE3) with two different MUC1 antibodies (NCRC11 and DF3) found the cytokeratin antibodies to be at least as good as the MUC1 antibodies for the detection of metastatic deposits (Galea *et al.*, 1991; Elson *et al.*, 1993). The decreased staining efficiency with the cytokeratin antibody in the present study appears to be related to loss of expression of cytokeratins or to difficulty of detection as, unlike mucin detection with BC2, the detection with MNF.116 requires a trypsinisation step to reveal these epitopes. Positive control breast cancer sections were included in each staining run and were always positive using MNF.116. Staining of primary

**Table V** Number of cases with occult metastases detected at each of four 100 μm sectioning levels and with each of three staining techniques in 208 cases of axillary node-negative breast cancer

| | *Individual sectioning level* | | | | *Combined sectioning levels* | | | |
|---|---|---|---|---|---|---|---|---|
| *Stain* | *1* | *2* | *3* | *4* | *1, 2, 3 and 4* | *1 and 2* | *1 and 3* | *1 and 4* |
| H&E | 7 | 11 | 11 | 13 | 17 | 11 | 12 | 17 |
| MNF.116 | 27 | 26 | 25 | 25 | 37 | 32 | 33 | 33 |
| BC2 | 36 | 34 | 35 | 38 | 52 | 40 | 44 | 51 |
| Total[a] | 38 | 37 | 37 | 39 | 53 | 43 | 47 | 51 |

[a]Positive using at least one stain.

**Table VI** Univariate survival analyses in patients with axillary node-negative breast cancer separated on the basis of detection of metastases using a combination of immunohistochemical detection and step-sectioning, histological type, tumour size and histological and nuclear grade

| | | *Disease-free survival* | | *Overall survival* | |
|---|---|---|---|---|---|
| *Variable* | *Classifications* | $\chi^2$ | *P* | $\chi^2$ | *P* |
| Occult metastases | Absent, present* | 7.27 | 0.007 | 5.31 | 0.021 |
| Number of nodes involved | 0, 1*, >1* | 7.28 | 0.007 | 4.53 | 0.033 |
| Size of occult metastases | None, <0.5 mm*, >0.5 mm* | 8.80 | 0.003 | 5.08 | 0.024 |
| Histological type | Ductal, lobular, other | 0.42 | 0.52 (NS) | 0.99 | 0.32 (NS) |
| Tumour size | <20 mm, >20 mm* | 7.47 | 0.006 | 3.23 | 0.07 (NS) |
| Histological grade | 1, 2*, 3* | 4.72 | 0.030 | 4.77 | 0.029 |
| Nuclear grade | 1, 2*, 3* | 7.03 | 0.008 | 8.65 | 0.003 |
| Age | <50 years, >50 years | 0.88 | 0.39 (NS) | 2.26 | 0.13 (NS) |
| Microvascular invasion | Absent, present | 0.35 | 0.55 (NS) | 0.22 | 0.64 (NS) |

The classifications with poorer survival are marked with an asterisk. Statistics: log-rank text, $\chi^2$ and *P*-value shown; NS, not significant.

| No. of metastases | 0 | 20 | 40 | 60 | 80 | 100 | 120 |
|---|---|---|---|---|---|---|---|
| 0 | 148 | 139 | 130 | 118 | 81 | 53 | 33 |
| 1 | 38 | 35 | 31 | 27 | 18 | 14 | 10 |
| >1 | 13 | 12 | 11 | 8 | 7 | 4 | 3 |

| No. of metastases | 0 | 20 | 40 | 60 | 80 | 100 | 120 |
|---|---|---|---|---|---|---|---|
| 0 | 148 | 146 | 138 | 128 | 91 | 55 | 34 |
| 1 | 38 | 37 | 36 | 34 | 23 | 16 | 10 |
| >1 | 13 | 12 | 12 | 11 | 8 | 6 | 4 |

| Size of metastases | 0 | 20 | 40 | 60 | 80 | 100 | 120 |
|---|---|---|---|---|---|---|---|
| None | 148 | 139 | 130 | 118 | 81 | 53 | 33 |
| ≤0.5 mm | 34 | 32 | 29 | 24 | 19 | 14 | 10 |
| >0.5 mm | 17 | 15 | 13 | 11 | 6 | 4 | 3 |

| Size of metastases | 0 | 20 | 40 | 60 | 80 | 100 | 120 |
|---|---|---|---|---|---|---|---|
| None | 148 | 146 | 138 | 128 | 91 | 55 | 34 |
| ≤0.5 mm | 34 | 33 | 33 | 30 | 23 | 17 | 11 |
| >0.5 mm | 17 | 16+ | 15 | 15 | 8 | 5 | 3 |

**Figure 2** Disease-free (**a**,**c**) and overall (**b**,**d**) survival curves for 199 axillary node-negative breast cancer patients classified according to the number of nodes with occult metastases (**a**,**b**) and the size of occult metastases (**c**,**d**). The tables at the base of each graph show the number of disease-free or surviving and non-censored patients at the end of each 20 month survival interval. Statistics: Log-rank test, $\chi^2$ and $P$-value shown.

tumours with MNF.116 has shown some evidence for both of these phenomenon using adjacent normal breast glands as a positive control. In some specimens the staining of primary tumours was weak and irregular as was staining of normal glands, whereas in others, staining in tumours was weak or absent despite strong reactivity in adjacent normal glands. However, it must also be noted that due to the complex nature of the MUC1 mucin and the influence of glycosylation on the reactivity of antibodies reactive with the MUC1 core protein, the reactivity of different antibodies with cancer-associated mucin can vary considerably, and the BC2 antibody may be superior to other antibodies previously used (McGuckin *et al.*, 1995).

The higher rate of detection of occult metastases in younger patients found in the present series is consistent with the higher rate of detection reported in the Ludwig group

**Table VII** Relative hazard rates for potential prognostic variables associated with disease-free and overall survival determined using Cox multivariate proportional hazards regression analyses

| *Variable* | *Relative hazard rates* *Disease-free survival* | *Overall survival* |
|---|---|---|
| *Presence/size of metastases* | | |
| Absent | 1.0 | 1.0 |
| <0.5 mm | 1.64 (0.73, 3.67) | 1.91 (0.73, 5.05) |
| >0.5 mm | 3.72 (1.43, 9.69) | 2.62 (1.43, 9.12) |
| *Histological grade* | | |
| 1 | 1.0 | 1.0 |
| 2 | 3.86 (1.11, 13.40) | 5.07 (0.86, 29.96) |
| 3 | 2.68 (0.63, 11.34) | 2.89 (0.41, 20.17) |
| *Tumour size* | | |
| <20 mm | 1.0 | 1.0 |
| >20 mm | 1.99 (1.02, 3.88) | 1.53 (0.65, 3.59) |
| *Nuclear grade* | | |
| 1 | 1.0 | 1.0 |
| 2 | 0.67 (0.19, 2.37) | 0.90 (0.14, 5.68) |
| 3 | 1.24 (0.33, 4.67) | 2.12 (0.33, 13.54) |

95% confidence intervals are shown in parenthesis.

| Index | 0 | 20 | 40 | 60 | 80 | 100 | 120 |
|---|---|---|---|---|---|---|---|
| 0 | 35 | 34 | 33 | 32 | 22 | 11 | 11 |
| 1 | 78 | 76 | 69 | 64 | 41 | 26 | 18 |
| 2 | 52 | 46 | 43 | 34 | 28 | 22 | 12 |
| 3 | 25 | 23 | 20 | 16 | 14 | 12 | 8 |
| 4 | 5 | 3 | 3 | 3 | 1 | 0 | 0 |

**Figure 3** Disease-free survival curve for axillary node-negative breast cancer patients classified according to the score of an index based on tumour size, histological grade and the presence/size of occult metastases. The table at the base of the graph shows the number of disease-free and non-censored patients at the end of each 20 month survival interval. Statistics: log-rank test, $\chi^2$ and *P*-value shown.

study (12% in women less than 50 *vs* 7% in women older than 50), [International (Ludwid) Breast Cancer Study Group, 1990]. A greater detection in younger women is consistent with a higher rate of detection of nodal metastases in younger women using standard histopathological techniques and is probably related to the generally more aggressive nature of breast cancers in these women. However, no relationship between metastases and age was demonstrated in two large studies using immunohistochemical detection (Hainsworth *et al.*, 1993; Nasser *et al.*, 1993). The higher rate of detection of occult metastases in lobular compared with ductal carcinoma confirms the findings of Trojani *et al.*, (1987*a*). Metastatic lobular carcinoma appears more likely to present as occult deposits of scattered cells within the nodal parenchyma than metastatic ductal carcinoma. In the present study these scattered deposits were often quite large and they obviously present a diagnostic difficulty using H&E due to morphological similarities with surrounding cells in the lymph node. Consistent with this hypothesis, the large Ludwig group study based on serial sectioning and H&E detection showed an equivalent rate of detection of metastases from IDC and ILC.

The results of this study clearly show poorer disease-free and overall survival in patients with occult metastases. These data also support the findings of previous studies using immunohistochemical detection that showed that the size of occult metastases and number of nodes involved have prognostic importance (Hainsworth *et al.*, 1993; Nasser *et al.*, 1993). Although the number of cases of ILC in this study is quite small, the survival results from this group do not contradict the findings of a previous study with more patients with ILC that found that occult metastases had little prognostic significance in ILC (Trojani *et al.*, 1987*a*). The differences in survival between patients with ILC and IDC who also have occult metastases may reflect biological differences consistent with their different patterns of metastatic spread characterised by Harris *et al.* (1984). The independent significance of the presence/size of occult metastases suggests that this factor could be combined with other more traditionally used prognostic factors when assessing the risk of recurrence or death of patients with apparently node-negative breast cancer. The simple index created from presence/size of occult metastases, histological grade and tumour size suggests that these patients could well be stratified into prognostic groups that may be used for determining and tailoring adjuvant systemic treatments in this group of patients traditionally regarded as node negative and in whom the selection of patients truly requiring systemic treatment has formerly been difficult. It may be that in the future that such an index including detection of occult metastases could be combined with biological markers of poor prognosis such as alterations in tumour-suppressor genes and increased expression of oncogenes. We shall be examining the associations of such factors in these patients in the near future. Detection of metastatic tumour cells in bone marrow of breast cancer patients is now also feasible and may have prognostic significance (Cote *et al.*, 1991; Dearnaley *et al.*, 1991), and could also be combined into a risk index.

We have demonstrated a high rate of occult metastases in patients with ANN breast cancer and have shown that these metastases can be detected using practical and affordable variations to standard histopathological techniques. The presence of these metastases is an independent indicator of poor prognosis and therefore should be considered for inclusion into indices used to stratify ANN patients into risk groups for consideration for adjuvant therapy.

**Acknowledgements**
This research was supported by grants from the Queensland Cancer Fund and the Royal Brisbane Hospital Private Practice Trust Fund. Dr McGuckin was supported by a grant from the National Health and Medical Research Council of Australia. Our gratitude is extended to Dianna Battistuta for statistical advice and particularly for performing the Cox multivariate regression analyses.

### References

ACKERMAN LV AND ROSAI HJ. (1974). *Surgical Pathology*, 5th Edn, pp 895–896. CB Mosby: St Louis.

BYRNE J, HORGAN PG, ENGLAND S, CALLAGHAN J AND GIVEN HF. (1992). A preliminary report on the usefulness of monoclonal antibodies to CA 15-3 and MCA in the detection of micrometastases in axillary lymph nodes draining primary breast carcinoma. *Eur. J. Cancer*, **28**, 658–660.

COTE RJ, ROSEN PP, LESSER ML, OLD LJ AND OSBORNE MP. (1991). Prediction of early relapse in patients with operable breast cancer by detection of occult bone marrow micrometastases. *J. Clin. Oncol.*, **9**, 1749–1756.

DE MASCAREL I, BONICHON F, COINDRE JM AND TROJANI M. (1992). Prognostic significance of breast cancer axillary lymph node micrometastases assessed by two special techniques: reevaluation with longer follow-up. *Br. J. Cancer*, **66**, 523–527.

DEARNALEY DP, ORMEROD MG AND SLOANE JP. (1991). Micrometastases in breast cancer: long-term follow-up of the first patient cohort. *Eur. J. Cancer*, **27**, 236–239.

ELSON CE, KUFE D AND JOHNSTON WW. (1993). Immunohistochemical detection and significance of axillary lymph node micrometastases in breast carcinoma. A study of 97 cases. *Anal. Quant. Cytol. Histol.*, **15**, 171–178.

ELSTON CW AND ELLIS IO. (1991). Pathological prognostic factors in breast cancer. I. The value of histological grade in breast cancer: experience from a large study with long-term follow-up. *Histopathology*, **19**, 403–410.

FISHER B AND REDMOND C. (1992). Systemic therapy in node-negative patients: updated findings from NSABP clinical trials. National Surgical Adjuvant Breast and Bowel Project. *Monogr. Natl Cancer Inst.*, **11**, 105–116.

FRIEDMAN S, BERTIN F, MOURIESSE H, BENCHABAT A, GENIN J AND SARRAZIN D. (1988). Importance of tumor cells in axillary node sinus margins ('clandestine' metastases) discovered by serial sectioning in operable breast carcinoma. *Acta Oncol.*, **27**, 483–487.

GALEA MH, ATHANASSIOU E, BELL J, DILKS B, ROBERTSON JF, ELSTON CW, BLAMEY RW AND ELLIS IO. (1991). Occult regional lymph node metastases from breast carcinoma: immunohistological detection with antibodies CAM 5.2 and NCRC-11. *J. Pathol.*, **165**, 221-227.

HAINSWORTH PJ, TJANDRA JJ, STILLWELL RG, MACHET D, HENDERSON MA, RENNIE GC, MCKENZIE IFC AND BENNETT RC. (1993). Detection and significance of occult metastases in node-negative breast cancer. *Br. J. Surg.*, **80**, 459–463.

HARRIS M, HOWELL A, CHRISSOHOU M, SWINDELL RIC, HUDSON M AND SELLWOOD RA. (1984). A comparison of the metastatic pattern of infiltrating lobular carcinoma and infiltrating ductal carcinoma of the breast. *Br. J. Cancer*, **50**, 23–30.

INTERNATIONAL (LUDWIG) BREAST CANCER STUDY GROUP. (1990). Prognostic importance of occult axillary lymph node micrometastases from breast cancers. *Lancet*, **335**, 1565–1568.

MCGUCKIN MA, WALSH MD, HOHN BG, WARD BG AND WRIGHT RG. (1995). Prognostic significance of MUC1 epithelial mucin expression in carcinoma of the breast. *Hum. Pathol.*, **26**, 432–439.

MCGUIRE WL, TANDON AK, ALLRED DC, CHAMNESS GC, RAVDIN PM AND CLARK GM. (1992). Treatment decisions in axillary node-negative breast cancer patients. *Monogr. Natl Cancer Inst.*, 173–180.

NASSER IA, LEE AKC, BOSARI S, SAGANICH R, HEATLEY G AND SILVERMAN ML. (1993). Occult axillary lymph node metastases in node-negative breast carcinoma. *Hum. Pathol.*, **24**, 950–957.

NEMOTO T, VANA J, BEDWANI RN, BAKER HW, MCGREGOR FH AND MURPHY GP. (1980). Management and survival of female breast cancer: results of a national survey by the American College of Surgeons. *Cancer*, **45**, 2917–2924.

NIO Y, ZIGHELBOIM J, BEREK JS AND BONAVIDA B. (1990). Sensitivity of ovarian tumor cells to effector cells generated by various biological response modifiers. *Nat. Immun. Cell Growth Regul.*, **9**, 283–296.

NOGUCHI S, AIHARA T, NAKAMORI S, MOTOMURA K, INAJI H, IMAOKA S AND KOYAMA H. (1994). The detection of breast carcinoma micrometastases in axillary lymph nodes by means of reverse transcriptase-polymerase chain reaction. *Cancer*, **74**, 1595–1600.

RAYMOND WA AND LEONG AS-Y. (1989). Immunoperoxidase staining in the detection of lymph node metastases in stage 1 breast cancer. *Pathology*, **21**, 11–15.

STEWART HJ. (1992). The Scottish trial of adjuvant tamoxifen in node-negative breast cancer. Scottish Cancer Trials Breast Group. *Monogr. Natl Cancer Inst.*, **11**, 117–120.

TROJANI M, DE MASCAREL I, BONICHON F, COINDRE JM AND DELSOL G. (1987*a*). Micrometastases to axillary lymph nodes from carcinoma of breast: detection by immunohistochemistry and prognostic significance. *Br. J. Cancer*, **55**, 303–306.

TROJANI M, DE MASCAREL I, COINDRE JM AND BONICHON F. (1987*b*). Micrometastases to axillary lymph nodes from invasive lobular carcinoma of breast: detection by immunohistochemistry and prognostic significance. *Br. J. Cancer*, **56**, 838–839.

WELLS CA, HERYET A, BROCHIER J, GATTER KC AND MASON DY. (1984). The immunocytochemical detection of axillary micrometastases in breast cancer. *Br. J. Cancer*, **50**, 193–197.

WILKINSON EJ AND HAUSE L. (1974). Probability in lymph node sectioning. *Cancer*, **33**, 1269–1274.

WILKINSON EJ, HAUSE LL, HOFFMAN RG, KUZMA JF, ROTHWELL DJ, DONEGAN WL, CLOWRY LJ, ALMAGRO UA, CHOI H AND RIMM AA. (1982). Occult axillary lymph node metastases in invasive breast carcinoma: characteristics of the primary tumor and significance of the metastases. *Pathol. Annu.*, **17**, 67–91.

XING PX, TJANDRA JJ, STACKER SA, TEH JG, THOMPSON CH, MCLAUGHLIN PJ AND MCKENZIE IFC. (1989). Monoclonal antibodies reactive with mucin expressed in breast cancer. *Immunol. Cell Biol.*, **67**, 183–195.

XING PX, REYNOLDS K, TJANDRA JJ, TANG XL AND MCKENZIE IF. (1990). Synthetic peptides reactive with anti-human milk fat globule membrane monoclonal antibodies. *Cancer Res.*, **50**, 89–96.